# Efficiency of transplacental transfer of respiratory syncytial virus (RSV) specific antibodies among pregnant women in Kenya

**DOI:** 10.12688/wellcomeopenres.17636.1

**Published:** 2022-02-08

**Authors:** Joyce U. Nyiro, Elizabeth Bukusi, Dufton Mwaengo, Amek Nyaguara, Bryan Nyawanda, Nancy Otieno, Godfrey Bigogo, Nickson Murunga, Marc-Alain Widdowson, Jennifer R. Verani, Sandra S. Chaves, Hope Mwangudza, Calleb Odundo, James A. Berkley, D James Nokes, Patrick K. Munywoki

**Affiliations:** 1 Centre for Geographic Medicine Research-Coast, Kenya Medical Research Institute (KEMRI)-Wellcome Trust Research Programme, Kilifi, Kenya; 2 Centre for Microbiology Research, Kenya Medical Research Institute (KEMRI), Nairobi, Kenya; 3 Institute of Tropical and Infectious Diseases, University of Nairobi, Nairobi, Kenya; 4 Centre for Global Health Research, Kenya Medical Research Institute (KEMRI), Kisumu, Kenya; 5 Division of Global Health Protection, CDC-Kenya, U.S Centers for Disease Control and Prevention (CDC), Nairobi, Kenya; 6 Institute of Tropical Medicine, Antwerp, Belgium; 7 Influenza Division, U.S. Centers for Disease Control and Prevention (CDC), Atlanta, USA; 8 School of Life Sciences and Zeeman Institute (SBIDER), University of Warwick, Coventry, UK

**Keywords:** Pregnant women; transplacental transfer efficiency; Respiratory Syncytial Virus; Neutralising antibody, Maternal vaccine, Effectiveness

## Abstract

**Background:** Maternal immunisation to boost respiratory syncytial virus (RSV) antibodies in pregnant women, is a strategy being considered to enhance infant protection from severe RSV associated disease. However, little is known about the efficiency of transplacental transfer of RSV-specific antibodies in a setting with a high burden of malaria and HIV, to guide the implementation of such a vaccination program.

**Methods:** Using a plaque reduction neutralization assay, we screened 400 pairs of cord and maternal serum specimens from pregnant women for RSV-specific antibodies. Participants were pregnant women of two surveillance cohorts: 200 participants from a hospital cohort in Kilifi, Coastal Kenya and 200 participants from a surveillance cohort in Siaya, Western Kenya. Transplacental transfer efficiency was determined by the cord to maternal transfer ratio (CMTR). Logistic regression was used to determine independent predictors of impaired transplacental transfer of RSV-specific antibodies.

**Results:** A total of 800 samples were screened from the 400 participants. At enrollment the median age was 25 years (Interquartile range (IQR): 21-31). Overall, transplacental transfer was efficient and did not differ between Kilifi and Siaya cohort (1.02 vs. 1.02; p=0.946) but was significantly reduced among HIV-infected mothers compared to HIV-uninfected mothers (mean CMTR: 0.98 vs 1.03; p=0.015). Prematurity <33 weeks gestation (Odds ratio [OR]: 0.23, 95% confidence interval [CI] 0.06–0.85; p=0.028), low birth weight <2.5 kgs (OR: 0.25, 95% CI: 0.07–0.94; p=0.041) and HIV infection (OR: 0.47, 95% CI:0.23-0.98; p=0.045) reduced efficiency of transplacental transfer among these women.

**Conclusions: **Transplacental transfer of RSV-specific antibodies among pregnant women in Kenya is efficient. A consideration to integrate other preventive interventions with maternal RSV vaccination targeting infants born premature (<33 weeks gestation), with low birth weight <2.5 kgs, or HIV-infected mothers is likely to improve vaccine outcomes in this setting.

## Abbreviations

RSV         Respiratory syncytial virus

KHDSS    Kilifi Health and Demographic Surveillance System

KEMRI    Kenya Medical Research Institute

KWTRP   KEMRI Wellcome Trust Research Programme

KCH         Kilifi County Hospital

LMICs      Low- and Middle -Income Countries

SD            Standard deviation

95% CI     95% Confidence interval

CMTR     Cord to Maternal Transfer Ratio

## Introduction

Globally, respiratory syncytial virus (RSV) is a significant cause of acute lower respiratory tract infection (LRTI) among infants leading to hospital admissions and in-hospital deaths, with 99% of these deaths occurring in developing countries ^1^. In sub-Saharan Africa and Asia, RSV has been observed to be responsible for about 40% of all hospital admissions with severe or very severe pneumonia among infants under 1 year ^2^. Severe RSV-associated LRTI is most common among infants under six months of age ^3, 4^, resulting in about 32% of hospitalised infants in the rural coast of Kenya, during epidemics ^3^.

Maternal immunisation is currently being considered as a strategy to protect infants from severe RSV-associated disease because of the lack of licenced RSV vaccines targeting infants. Additionally, the frequency of cases and the peak hospitalization occurs at 2 months of age which is less than the timing for first scheduled infant immunisations and during this neonatal period, the infant immune system is not mature enough to mount protective immunity ^5– 8^. Efforts to advance maternal immunisation for RSV have shown promise and several candidate maternal RSV vaccines are in the late stages of clinical trials ^8– 10^. However, despite the advancement in development of maternal RSV vaccines, the success of this program will depend on how efficiently vaccine-induced RSV-specific antibodies are transferred to the infant.

Previous studies have shown that transplacental transfer of RSV-specific antibody to the infant is usually efficient with a cord blood to maternal blood antibody titre ratio of ≥ 1 ^11, 12^. Transplacental transfer of IgG antibodies begins during the 28 ^th^ week of gestation which is coincident with the timing for expression of Fc gamma RII (FcgII) receptor responsible for the materno-foetal transfer of antibodies ^13^. Thus, infants born preterm, shortly after or before initiation of transplacental transfer of antibodies are less likely to benefit from a maternal RSV vaccine program. Placental malaria, hypergammaglobinaemia (total IgG >15g/L), HIV infection and possible illness episodes or infection occurring during the third trimester of pregnancy have been known to influence the level of antibodies transferred to the infant ^14– 16^. However, there is limited data on the efficiency of transplacental transfer of RSV-specific antibodies in settings where the maternal population experiences comorbidities such as malaria, HIV and undernutrition as well as premature deliveries, which could negatively impact the effectiveness of a maternal RSV vaccine.

In this study, we describe the efficiency of transplacental transfer of RSV-specific antibodies among pregnant women in Kenya using cord-maternal blood sample pairs collected from pregnant women in the counties of Kilifi (coast region) and Siaya (western region). We also describe background factors and illness episodes occurring during pregnancy that could influence transplacental transfer of RSV-specific antibodies to support the successful implementation of a maternal RSV vaccine program in Kenya.

## Methods

### Study sites and population

Data collection for this study was conducted at Kilifi County, Siaya and Bondo areas in Siaya County, Kenya. The study population are pregnant women who were participants in two separate cohort studies; a hospital-based surveillance activity investigating risk factors for severe morbidity and mortality in mothers and their infants in Kilifi, coastal Kenya, and a second in Siaya County, Western Kenya, for surveillance of influenza disease.

The surveillance to investigate risk factors for severe morbidity and mortality in mothers and their infants was set up by KEMRI-Wellcome Trust Research Programme (KWTRP) at the maternity ward of Kilifi County Hospital (KCH) and the Kilifi Health and Demographic Surveillance System (KHDSS) area in Coastal Kenya in 2011 ^17^. This surveillance was designed to observe 4600 births with approximately 2300 being residents of KHDSS. All mothers presenting for delivery at the maternity ward of KCH were invited to enroll. Routine clinical data were collected using a standardized questionnaire at admission to the maternity department and following delivery. During this surveillance, consent was sought from pregnant women presenting at KCH maternity ward to collect cord and maternal blood samples after delivery. These samples were securely stored at -80˚C at KEMRI-Wellcome Trust laboratories in Kilifi for molecular and serological testing for viral and bacterial pathogens. The KIPMAT surveillance enrolled 4047 pregnant women in 2018 and 2019 who had a cord and maternal blood samples collected at delivery and stored for future use.

From the Western part of Kenya, in Siaya County, a cohort of pregnant women was set up through a collaboration between KEMRI-Centre for Global Health Research and the U.S. Centers for Disease Control and Prevention (CDC) Kenya in 2015. This surveillance included pregnant women recruited either from their homes or when they visited for antenatal care at Bondo sub-County or Siaya County Referral Hospital. Participants were enrolled at gestational age <20 weeks. These pregnant women were followed up weekly through a phone call or home visit to record any occurrence of influenza-like illness episodes. Blood samples were collected at enrolment and a maternal and cord blood at birth. If a pregnant woman was identified with cough or fever during follow up, a respiratory specimen was collected and screened for influenza virus type A and B and for RSV using molecular methods ^18^. All participants were requested to deliver their children in the hospital where birth outcomes were recorded; thereafter, both the baby and the mother were followed up weekly for up to six months post-delivery to assess infection from respiratory viruses by testing nasal and throat swabs from symptomatic cases by RT-PCR.

During the RSV RT-PCR procedure, nucleic acids were extracted from 100ul of the combination of nasopharyngeal and oropharyngeal specimens using the MagMAX™ Viral RNA Isolation Kit on the Kingfisher mL platform (Life Technologies, New York). A 5ul of the Nucleic acid extract was then tested for RSV in a 1-step real-time reverse-transcription polymerase chain reaction (rRT-PCR) assay, using the AgPath-ID One-step RT-PCR kit (Applied Biosystems, Foster City, California) using CDC’s primers and fluorescent-labelled hydrolysis probes. The assay was considered positive for RSV when exponential fluorescence curves crossed the assigned threshold at a cycle threshold value of <37.0.

The influenza surveillance had initially proposed to recruit 2250 pregnant women in a period of 3–5 years from 2015, based on assumptions that, each participant was to be followed up for a period of six months during pregnancy and 15% of participants were expected to have at least one influenza-like illness (ILI) episode. The study changed to a surveillance in 2018 and continued to enrol participants beyond the proposed sample size. From 2018 to 2019, the influenza surveillance had recruited 1458 pregnant women from whom, 795 participants had a cord and maternal blood samples at delivery, and these were used as a sampling frame for this study.

A computer-generated random sample of 400 participants (200 from each region) was selected from the cohort registers based on the availability of meta-data and paired cord and maternal blood samples for births (including preterm births) that occurred in 2018 and 2019. The sample size was estimated using Edgar C. Fieller methods of calculating confidence intervals for the ratio of two means. This sample size method used cord-to-maternal transfer ratio (CMTRs) of 1.03 (0.88–1.19) observed in women in rural Nepal ^19^. Assuming that CMTR of RSV-specific antibodies among women in Kenya were similar to those of Nepalese women and both the cord and maternal antibody levels followed a Gaussian distribution, a sample size of 200 mother-infant pair was sufficient to detect a CMTR of 1.03 with a 95% confidence interval of 1.01–1.06.

### Laboratory procedures

All blood samples were screened for RSV specific antibodies using an inhouse plaque reduction neutralization titre (PRNT) assay ^20, 21^ at KWTRP laboratories, Kilifi, Kenya. The PRNT procedure determines the concentration of functional antibodies from a human serum sample (or antibody preparation) required to induce 50% neutralization of a known titration of RSV virus using the Spearman Karber method ^22^. In this assay, micro RSV plaques are stained brown by immunoperoxidase and counted with an ELISPOT reader. This assay has been described elsewhere in detail ^21^.

An RSV group A human reference standard (RSV IS 16/284) ^23^ obtained from National Institute for Biological Standards and Control (NIBSC), Potters Bar, UK, and an inhouse pooled adult sera were incorporated into each assay run to check for antibody deterioration, standardization of sample titres and quality control.

### Ethical considerations

Written informed consent to collect samples and data for storage and use in other studies was obtained from all participants through the parent studies, i.e., the influenza cohort surveillance (SERU #2880; CDC IRB number 6709) and the surveillance for risk factors cohort (SERU #1778). Ethical approval to screen samples for RSV-specific antibodies and use of data from the parent studies for this study was granted by the KEMRI Scientific and Ethical Review Unit Committee (SERU #3716). All methods were carried out in accordance with relevant guidelines and regulations.

### Statistical analyses

Separate analysis was done for each cohort and with combined data from both cohorts. The efficiency of transplacental transfer of RSV-specific antibodies was calculated for each mother-infant pair of blood samples. A CMTR (i.e., PRNT titre cord/PRNT titre maternal blood) of ≥1 was considered normal or efficient, CMTR <1 but ≥0.8 as impaired and <0.8 as severely impaired or poor. Duration of transplacental transfer was calculated as gestational age at delivery minus 28; where 28 ^th^ week was estimated as the gestational age when a transplacental transfer of IgG antibodies begins during pregnancy. For this analysis, Preterm birth (PTB) was defined as baby born alive before 37 weeks of pregnancy are completed and very early PTB as baby born <33 weeks of gestation. The difference in CMTR, cord or maternal RSV PRNT titres between HIV-infected versus HIV-uninfected mothers and RSV-infected infants vs. uninfected infants were analysed using a two-sample paired t-test. The Chi-square test was used to compare characteristics of women between Kilifi and Siaya cohort; and was also applied to determine the association between maternal/infant characteristics (HIV infection, malaria infection, RSV infection, anaemia, education level, occupation, gestational age at delivery and birth weight) and efficiency of transplacental transfer of RSV-specific antibodies. Logistic regression adjusted for each variable category (HIV infection, malaria infection, gestational age at delivery, gravida, birthweight and RSV infection during pregnancy) was used to determine independent predictors of an impaired transplacental transfer of RSV specific antibodies. All data analysis was conducted using STATA version 15.0 (Stata Corp, College Station, Texas). However, a R Statistical software version 4.1.1 which is on open-access, can perform the equivalent analysis. To replicate the same analysis in R, we advise the user to import the CSV version of the data and follow the steps provided in the STATA do-file.

## Results

### Characteristics of study participants

A total of 800 cord and maternal blood samples from 400 participants selected from the two cohorts were screened for RSV-specific neutralizing antibodies ^24^. The median age of the women at enrollment was 25 years (Interquartile range (IQR): 21–31 years). About 95% of these women reported being married, 6% had no formal education, 57% were housewives and 21% had experienced more than six live births ( Table 1).

**Table 1.** Characteristics of study participants from Kilifi and Siaya.

Characteristic	Kilifi (n)	%	Siaya (n)	%	Total (n)	%	P * value
	200	50	200	50	400	100	
**Maternal age**							
15-19	31	15.50	4	2.00	35	8.75	
20-24	65	32.50	81	40.50	146	36.50	
25-29	43	21.50	60	30.00	103	25.75	<0.001
30-34	30	15.00	32	16.00	62	15.50	
35-39	16	8.00	19	9.50	35	8.75	
40-44	15	7.50	2	1.00	17	4.25	
45-49	0	0.00	2	1.00	2	0.50	
**Marital status**							
Married	191	95.50	190	95.00	381	95.25	
Single	9	4.50	10	5.00	19	4.75	0.814
**Gestational age at delivery**							
<33 weeks	10	5.00	3	1.50	13	3.25	
33-37 weeks	59	29.50	44	22.00	103	25.75	0.022
38-42 weeks	131	65.50	153	76.50	284	71.00	
**Education level**							
None	23	11.50	2	1.00	25	6.25	
Primary	124	62.00	108	54.00	232	58.00	<0.001
Secondary	37	18.50	74	37.00	111	27.75	
Tertiary-College/University	16	8.00	16	8.00	32	8.00	
**Gravida**							
1-2	112	56.00	41	20.50	153	38.25	
3-5	65	32.50	127	63.50	192	48.00	<0.001
6-9	18	9.00	31	15.50	49	12.25	
10-15	5	2.50	1	0.50	6	1.50	
**Occupation**							
Farmer	1	0.50	13	6.50	14	3.50	
Business woman	23	11.50	59	29.50	82	20.50	
House wife	136	68.00	92	46.00	228	57.00	<0.001
Salaried worker	16	8.00	18	9.00	34	8.50	
Other	24	12.00	18	9.00	42	10.50	
**Number of ANC visits**							
1	9	4.52	146	73.00	155	38.85	
2	22	11.06	45	22.50	67	16.79	
3	45	22.61	7	3.50	52	13.03	<0.000
4	57	28.64	2	1.00	59	14.79	
5	38	19.10	0	0.00	38	9.52	
6	28	14.07	0	0.00	28	7.02	
**Sex of child**							
Female	94	47.00	96	48.00	190	47.50	0.841
**Birthweight**							
Underweight (<2.5kgs)	43	21.50	12	6.00	55	13.75	<0.000
**Transplacental transfer Efficiency**							
Impaired	77	38.50	89	44.50	166	41.50	0.223

(*P-Chi squared value)

The overall mean (SD) birth weight (from both cohorts) of infants was 3.03 kgs (0.56), and 55 (14%) of the infants were born with low birth weight <2.5 kilograms. The mean (SD) gestational age at delivery was 38.3 weeks (2.62). There were 11 infants out of 200 infants born from women sampled from the Siaya cohort who got RSV infection under 6 months of age. Additionally, among women from the Siaya cohort, 37 (19%) were HIV infected, 52 (26%) had malaria infection, 5 (3%) had RSV infection and 12 (6%) had severe anaemia during pregnancy. These additional data were not available for women from the Kilifi cohort.

Analysis of the difference in characteristics of participants from the two cohorts showed these women were significantly different in most characteristics ( Table 1). Compared to the Siaya sample, Kilifi pregnant women had more premature births with gestational age <33 weeks (5% vs. 2%; P=0.022), more babies were born with low birthweight <2.5 kgs (21.5% vs. 6%; P<0.001), more women had lower than the secondary level of formal education (73.5% vs. 55%; P<0.001), more were younger, i.e.19 years and below (16% vs. 2%; P<0.001), and more women were housewives (68% vs. 46%; P<0.001). However, these women were similar in proportion of those married (95.5% vs. 95%; P= 0.814) and female sex of the infant (47% vs. 48%; P=0.841).

### Distribution of Cord and Maternal RSV-specific antibodies among pregnant women in Kenya

**Figure 1.**
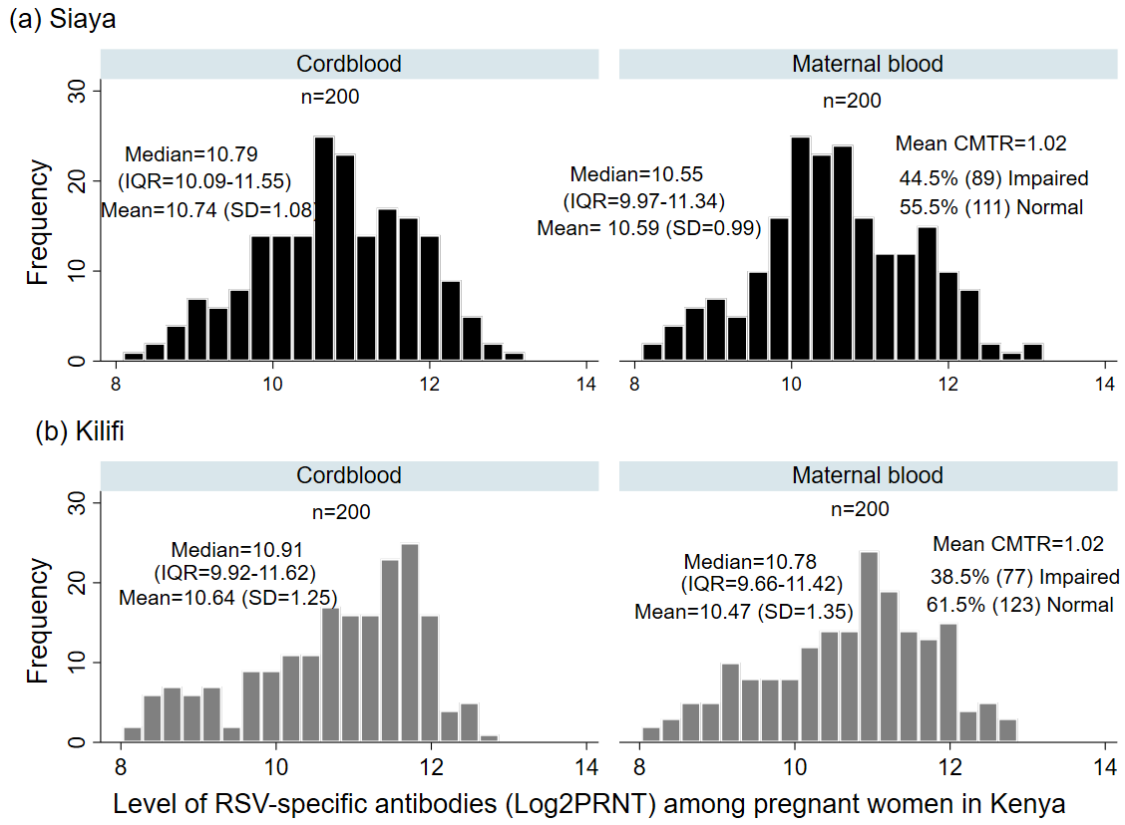
Frequency distribution of cord and maternal RSV specific antibodies (log2 transformed PRNT titres) at birth from 400 women from Kilifi and Siaya, Kenya. The mean (standard deviation), median (Inter quartile range) and the mean cord to maternal transfer ratio for each study cohort is shown.

Overall, the mean cord PRNT titres from both cohorts was 10.69 log _2_PRNT (SD: 1.17), with a median titre of 10.85 log _2_PRNT (IQR 10.00–11.59), while the mean maternal log _2_PRNT RSV antibodies was 10.53 log _2_PRNT (SD:1.19), with a median of 10.62 log _2_PRNT (IQR 9.92–11.40). The mean titres of cord RSV-specific antibodies from the Kilifi cohort was 10.64 log _2_PRNT (SD:1.25), with a median titre of 10.91 log _2_PRNT (Interquartile Range (IQR) 9.92 -11.62). In the Siaya cohort, the mean cord RSV antibody titre was 10.74 log _2_PRNT (SD:1.08), and median 10.79 log _2_PRNT (IQR 10.09–11.55) respectively ( Figure 1). Both mean cord (10.64 vs. 10.74; p=0.374) and mean maternal (10.47 vs. 10.59; p=0.319) log _2_PRNT titres of RSV-specific antibodies between Kilifi and Siaya cohorts were not significantly different.

For the Siaya cohort, we found no difference in the mean log _2_PRNT titres in cord blood for infants with RSV infection compared to infants without RSV infection in the first 6 months of life (10.50 log _2_PRNT (SD: 0.99) vs. 10.74 (SD: 1.07) respectively, p=0.186). The mean (SD) cord PRNT titres transferred to infants of 37 (19%) HIV-infected mothers of 10.41 (SD: 1.14) log _2_PRNT was significantly lower (p=0.039) than that of infants from 163 HIV-uninfected mothers 10.81 (SD: 1.05) log _2_PRNT. The cord log _2_PRNT RSV antibody levels of infants from mothers with and without severe anaemia (10.87 vs. 10.73: p=0.202) and mothers with and without malaria (10.90 vs. 10.68: p=0.666) were not significantly different.

### Efficiency of Transplacental Transfer of RSV-specific antibodies

The transplacental transfer of RSV-specific antibodies among these women was efficient with a mean CMTR of 1.02 (SD=0.09) and median 1.01 (IQR 0.97–1.06). The mean CMTR values were similar for pregnant women from Kilifi and Siaya (1.02 vs. 1.02; p=0.946; t=0.067).

The transplacental transfer of RSV specific antibodies for women from Kilifi and Siaya cohort was severely impaired in 1 (0.5%) and 6 (3.0%), impaired in 76 (38.0%) and 83 (41.5%), and normal in 123 (61.5%) and 111 (55.5%), respectively. The overall proportion of women from the two cohorts with impaired transplacental transfer was 41.5% (166/400; 77 Kilifi vs. 89 Siaya).

Analysis of the trend in efficiency of transplacental transfer and characteristics of these women ( Table 2), showed a significantly lower CMTR value among women who were HIV-infected (mean CMTR: 0.98 vs. 1.03; p=0.015) and women who reported their occupation as farming (mean CMTR: 0.96 vs.1.02; p=0.012). The CMTR value among women who got RSV infection during pregnancy (mean CMTR: 0.98 vs. 1.02; p=0.416) and that of mothers whom infants got RSV disease under 6 months of age (1.01 vs. 1.02; p=0.489) was not different.

**Table 2.** Transplacental transfer of RSV specific antibodies provided as cord to maternal transfer ratio (CMTR) with characteristics of women from Kilifi and Siaya, Kenya.

	Efficiency of Transplacental Transfer of RSV specific antibodies								
Characteristic	Kilifi			Siaya			All women		
	n=200	Mean CMTR	SD*	n=200	Mean CMTR	SD*	n =400	Mean CMTR	SD*
**Gestational age at delivery**									
<33 weeks	10	1.00	0.08	2	0.90	0.06	13	0.98	0.09
34–37 weeks	59	1.02	0.06	44	1.02	0.08	103	1.02	0.07
38–42 weeks	131	1.02	0.07	153	1.02	0.11	284	1.02	0.10
**Gravida**									
1–2	112	1.02	0.07	41	1.05	0.10	153	1.03	0.08
3–5	65	1.01	0.08	127	1.02	0.11	192	1.02	0.10
6–9	18	1.00	0.07	31	0.98	0.08	49	0.99	0.08
10–15	5	0.97	0.11	1	0.99		6	0.98	0.10
**Occupation**									
Farmer	1	0.98		13	0.96	0.06	14	0.96	0.06
Business woman	23	1.03	0.07	59	1.02	0.09	82	1.02	0.09
Housewife	136	1.01	0.07	92	1.02	0.11	228	1.02	0.09
Salaried worker	16	1.03	0.07	18	1.03	0.14	34	1.04	0.11
Other	24	1.02	0.08	18	1.04	0.06	42	1.03	0.07
**Marital status**									
Single	9	1.01	0.06	10	0.99	0.11	19	1	0.08
Married	191	1.02	0.07	190	1.02	0.1	381	1.02	0.09
**Maternal age (yrs)**									
16–19	31	1.02	0.07	4	0.96	0.21	35	1.02	0.09
20–29	108	1.02	0.07	141	1.02	0.10	249	1.02	0.09
30–39	46	1.02	0.07	51	1.00	0.10	97	1.01	0.09
40–49	15	1.03	0.08	4	0.94	0.06	19	1.00	0.09
**Education level**									
None	23	1.01	0.08	2	1.06	0.04	25	1.01	0.08
Primary	124	1.01	0.07	108	1.02	0.09	232	1.02	0.08
Secondary	37	1.03	0.07	74	1.01	0.13	111	1.02	0.12
Tertiary	16	1.05	0.09	16	1.03	0.05	32	1.04	0.08
**HIV status**									
Negative				163	1.03	0.11			
Positive				37	0.98	0.08			
**Severe anaemia**									
No				188	1.02	0.11			
Yes				12	1.00	0.07			
**Malaria**									
No				148	1.01	0.11			
Yes				52	1.04	0.08			
**Maternal RSV infection**									
Negative				195	1.02	0.11			
Positive				5	0.98	0.86			

### Illness episodes during pregnancy and transplacental transfer of RSV-specific antibodies

Assessment of illness episodes from the 200 women sampled from the Siaya cohort showed 120 (60%) pregnant women had sick outpatient visits captured during weekly follow-ups and 6 of them required hospitalisation. Data on illness episodes during pregnancy among the Kilifi women was not available for this study because the surveillance was not designed to follow up participants prior to delivery.

The most common complaints about the outpatient visits among Siaya women were cough 67 (55.8%), abdominal pain 67 (55.8%), other acute respiratory illness (runny nose, shortness in breathing and chest pain while breathing 35 (29.2%)), joint pain 27 (22.5%), vomiting or diarrhoea 22 (18.3%), urinary tract infection 18 (15.0%), fever 17 (14.2%) and sore throat 12 (10.0%). One participant had premature labour. Experiencing cough episodes during pregnancy was found to be associated with impaired transplacental transfer of RSV-specific antibodies (67.3% vs. 32.7%: Chi ^2^ p=0.027).

Multiple illness episodes during the third trimester of pregnancy occurred in 79/120 (66%) of the sick participants. There were none of the following illness episodes reported during pregnancy in this sample of women: gestational diabetes, hypertension or pre-eclampsia. No illness episode during the third trimester of pregnancy was found to be associated with the efficiency of transplacental transfer of RSV-specific antibodies.

### Gestational age at delivery and transplacental transfer of RSV-specific antibodies

**Figure 2.**
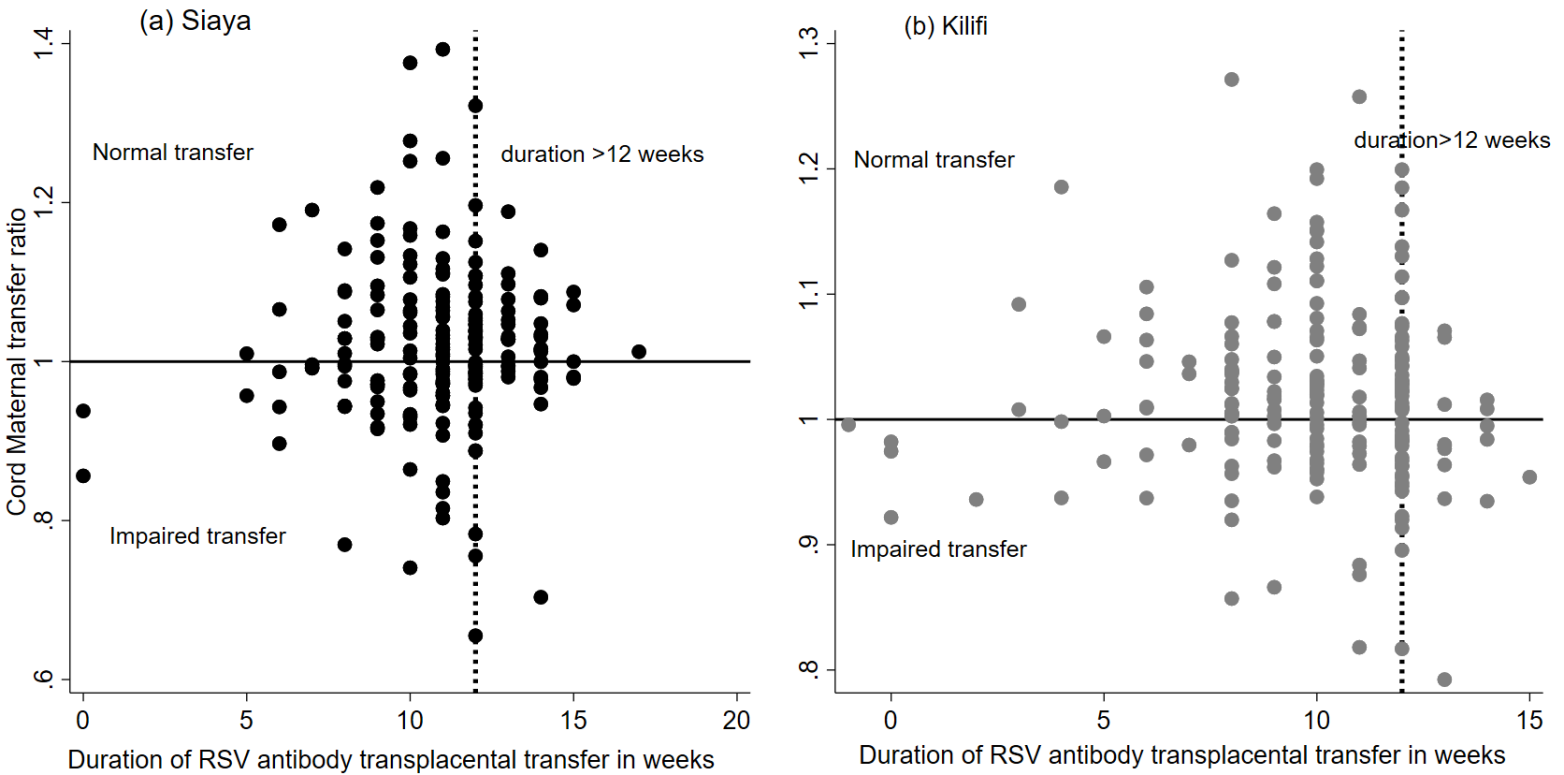
A scatter plot for cord to maternal transfer ratio by duration of transplacental transfer among pregnant women from Kilifi and Siaya, Kenya. The line for efficiency in each cohort is shown.

The effect of gestational age in influencing transplacental transfer of RSV antibodies is demonstrated in a scatterplot of CMTR by duration in weeks of transplacental transfer ( Figure 2). The graph shows the level of CMTR is less than 1 or impaired within 4 weeks after onset of transplacental transfer (onset period estimated at 28 ^th^ week of gestation); CMTR gradually increases above one in most participants in the next 8 weeks and starts to decline to low levels 12 weeks after onset of transplacental transfer.

### Factors influencing transplacental transfer of RSV-specific antibodies among pregnant women in Kenya

Among pregnant women in Kilifi, gestational age at delivery of <33 weeks was found to be significantly associated with reduced transplacental transfer of RSV-specific antibodies (p=0.034). In a univariate logistic analysis, transplacental transfer was likely to be increased 5.8 times more in births occurring between 34–37 weeks compared to births in less than 33 weeks of gestation (Odds ratio (OR): 5.8, 95% confidence interval (CI)1.33–24.95) ( Table 3).

**Table 3.** Predictors of an Impaired transplacental transfer among pregnant women from Kilifi cohort (univariant analysis).

		Efficiency of transplacental transfer (Kilifi)				Chi2				Odds ratio
Characteristic	Category	Normal		Impaired		P value	OR	Odds Ratio (95% CI)		P value
		n(123)	%	n(77)	%			LCL	UCL	
**Gestational age** ** delivery**										
	<33 weeks	3	2.4	7	9.1		Ref			
	34–37 weeks	42	34.2	17	22.1	**0.034**	5.8	1.33	24.95	0.019
	38–42 weeks	78	63.4	53	68.8		3.4	0.84	13.88	0.083
**Birthweight**										
	Underweight (<2.5kgs)	25	20.3	18	23.4					
	Normal	98	79.7	59	76.6	0.609				
**Maternal ** **age(yrs)**										
	16–19	18	14.6	13	16.9					
	20–29	65	52.9	43	55.8	0.589				
	30–39	32	26.0	14	18.8					
	40–49	8	6.5	7	9.1					
**Education level**										
	None	13	10.57	10	12.99					
	Primary	74	60.16	50	64.94	0.718				
	Secondary	25	20.33	12	15.58					
	Tertiary	11	8.94	5	6.49					
**Gravida**										
	1–2	72	58.54	40	51.0					
	3–5	41	33.33	24	31.2	0.305				
	6–9	8	6.5	10	13.0					
	10–15	2	1.63	3	3.9					
**On medication**										
	No	13	10.6	6	7.8					
	Yes	110	89.4	71	92.2	0.515				

In the Siaya cohort, transplacental transfer of RSV-specific antibodies was found to be significantly impaired among women with; gravida of more than 6 (OR: 0.56, 95% CI:0.35–0.91; p=0.02), occupation as farming (OR: 0.13, 95% CI:0.03–0.60; p=0.009), HIV infection (OR: 0.47, 95% CI:0.23–0.98; p=0.045) and infants with low birth weight <2.5 kilograms (kgs) (OR: 0.25, 95% CI:0.065–0.94; p=0.041). In a multivariate analysis including gravida, HIV infection, occupation and birthweight to the model for Siaya women, only low birth weight <2.5 kgs was strongly associated with reduced efficiency of transplacental transfer of RSV specific antibodies (OR: 0.21, 95% CI:0.05–0.85; p= 0.029). Malaria infection was significantly associated with increased transplacental transfer of RSV-specific antibodies (OR: 1.95, 95% CI:1.01–3.78; p=0.048) ( Table 4). The majority of pregnancies (76%) among Siaya women were delivered at term, i.e., ≥37 weeks and there was no significant association found between efficiency of transplacental transfer and gestational age at delivery (p=0.186).

**Table 4.** Predictors of an Impaired transplacental transfer among pregnant women from Siaya cohort (univariant analysis).

		Transplacental transfer efficiency (Siaya)				Chi2				Odds ratio
Characteristic	Category	Normal		Impaired		P value	OR	Odds Ratio (95%CI)		P value
		n(111)	%	n(89)	%			LCL	UCL	
**Maternal age (yrs)**	15-19	2	1.82	1	1.12					
	20-29	83	74.77	58	65.17					
	30-39	25	22.52	26	29.21	0.068				
	40-49	0	0.00	4	4.49					
**Gestational age at** ** delivery**	<33 weeks	0	0.00	2	2.25					
	34-37 weeks	22	19.82	22	24.72	0.186				
	38-42 weeks	88	80.18	65	73.03					
**Birthweight**										
	Underweight (<2.5kgs)	3	2.70	9	10.11					
	Normal	108	97.30	80	89.89	**0.028**	4.05	1.06	15.40	0.041
**Gravida**										
	1-2	27	24.32	14	15.73		Ref			
	3-5	72	64.86	55	61.80	**0.049**	0.67	0.33	1.42	0.301
	6-9	12	10.81	20	22.47		0.31	0.12	0.82	0.018
**Occupation**										
	Farmer	2	1.80	11	12.36		Ref			
	Business woman	37	33.33	22	24.72		9.25	1.87	45.65	0.006
	Housewife	50	45.05	42	47.19	**0.031**	6.55	1.37	31.21	0.018
	Salaried worker	10	9.01	8	8.99		6.87	1.17	40.38	0.033
	Other	12	10.81	6	6.74		11.00	1.82	66.37	0.009
**Education level**										
	Primary/None	57	51.35	53	59.55					
	Secondary	43	38.74	31	34.83	0.378				
	Tertiary	11	9.11	5	5.62					
**HIV status**										
	Negative	96	86.49	67	75.28		Ref			
	Positive	15	13.51	22	24.72	**0.043**	0.48	0.23	0.98	0.045
**Severe Anaemia**										
	No	106	95.50	82	92.13					
	Yes	5	4.50	7	7.87	0.320				
**Sick_Cough**										
	No	36	52.94	17	32.69		Ref			
	Yes	32	47.06	35	67.31	**0.027**	2.30	1.09	4.90	0.028
**Malaria**										
	No	76	68.47	72	80.90		Ref			
	Yes	35	31.53	17	19.10	**0.046**	1.95	1.00	3.78	0.048

In a multivariate logistic analysis of combined data from the Kilifi and Siaya cohorts, occupation as a farmer (OR: 0.16, 95% CI:0.03–0.73; p=0.018), gravida>6 (OR: 0.70, 95% CI:0.52–0.94; p=0.023) and gestational age at delivery <33 weeks (OR: 0.22, 95% CI:0.06–0.84; p=0.027) were significantly associated with reduced transplacental transfer of RSV-specific antibodies. Adjusting for the study site did not have any effect on these factors.

## Discussion

In this study, we found Kenyan women from the two geographical regions of Siaya and Kilifi differing in many characteristics, but these differences did not affect the mean levels of RSV-specific antibodies transferred to infants or the overall efficiency of transplacental transfer. We also found multiple factors, including gestational age less than 33 weeks, having had multiple pregnancies and farming as an occupation to be associated with reduced transplacental transfer of RSV specific antibodies among pregnant women from the two cohorts.

The concentration of RSV antibody transferred to infants by HIV-infected mothers were significantly reduced. Similarly, the trend of cord to maternal antibody transfer ratio showed a decrease with HIV infection. These findings are in line with previous studies ^25^ which together raise concerns involving the effectiveness of a maternal RSV vaccine introduction to low-and middle-income countries (LMICs), which is thought might be negatively impacted by the existing comorbidities. Ongoing clinical trials (NCT04424316; NCT04605159) ^8^ of maternal RSV vaccines are not taking into account HIV-diverse populations, or populations with high malaria prevalence. Therefore, investigating differences in transplacental transfer in these populations could be important in validating vaccine response in the future.

Interestingly, we found the concentration of RSV-specific antibodies of infants who got RSV infection within the first six months of life, to be lower than those without RSV infection, but not significantly different. This result is similar to a previous study conducted in rural Nepal ^19^ and a case control study done in Kilifi, Kenya ^26^, where cord antibody levels from wild-type RSV infection were found not to provide absolute protection against severe RSV associated disease. The CMTR values were also similar among these infants, implying that, the development of RSV disease was not necessarily influenced by the efficiency of transplacental transfer or levels of RSV-specific antibody, but possibly because antibodies from a wild type RSV infection lack palivizumab-competing antibody which have a strong neutralizing activity to both RSVA and RSVB ^27^. A RSV vaccine with high neutralizing activity should overcome this challenge.

In this study, gestational age at delivery <33 weeks showed reduced transplacental transfer of RSV-specific antibodies. Gestational age has been known to influence the transplacental transfer of IgG antibodies in the Gambia ^14^ and in Sri Lanka ^28^ where materno-foetal transfer of RSV-specific antibodies was impaired in premature babies. Furthermore, in estimating the duration of transplacental transfer by gestational age using the 28 ^th^ week of pregnancy as the onset for transplacental transfer, we found babies born shortly (<4 weeks) after the beginning of this transfer had an impaired transplacental transfer. Similarly, babies born more than 3 months after onset of transplacental transfer showed decreased CMTR which was as a result of antibody decay ^29^. Studies have also shown that, before the 26 ^th^ week of gestation, IgG transfer is blocked by a barrier of cytotrophoblasts under the syncytiotrophoblast layer ^30^ and Fc gamma RII (FcgII) receptors responsible for mediation of materno-foetal transfer of antibodies are not well expressed ^13^. Our results, therefore, demonstrate the phenomenon of accumulation of antibody concentration among infants with the time of transfer, waning of RSV-specific antibodies occurring with wild type RSV infection and confirm the influence of gestational age on timing for vaccination, likely to be observed during the implementation of a maternal RSV vaccine in this setting.

We also found having had more than 6 pregnancies, HIV infection, and low birth weight <2.5 kgs was associated with impaired transplacental transfer of RSV-specific antibodies. These results are similar to findings in the Gambia, where low birthweight <2.5 kgs was found to influence the transplacental transfer of RSV-specific studies ^14^. The role of HIV infection in impairing transplacental transfer of RSV antibodies has been found in studies conducted in Botswana ^31^ and Malawi ^32^. HIV-infected pregnant women have shown reduced immunogenicity to vaccines and this is thought to be related to immune activation leading to the production of inflammatory cytokines at the materno-foetal interface ^33, 34^. Women with multiple births or pregnancies are usually older and due to repeated exposure to RSV infection, they are thought to have an accumulation of higher levels of antibodies which causes saturation of Fc transport receptors ^35^ leading to reduced transfer and thereby low levels of antibodies observed in neonates.

Women diagnosed with malaria during pregnancy had an efficient transplacental transfer in this study contrary to what has been observed in other studies in Papua New Guinea and Malawi ^32, 36^ where malaria was associated with a decrease in the transplacental transfer of IgG antibodies of 81%. The process of materno-foetal transfer of pathogen specific antibodies is likely to vary between different populations due to impairment caused by saturation of these receptors with infection-related hypergammaglobineamia ^33^. We would therefore like to argue that, since antimalarial prophylaxis uptake is mandatory for all pregnant women in Kenya during antenatal visits, this might have played an important role in reducing malaria related hypergammaglobineamia. Thus, 67% of the women diagnosed with malaria infection were found to have a normal transplacental transfer of RSV-specific antibodies. However, further screening of samples for evidence of placental malaria is warranted among this sample of women.

This study has some limitations. First, we did not screen for total immuno-globulin G levels and thus are not able to confirm any infection related hypergammaglobulinemia to the impaired placental transfer of RSV antibody seen in HIV-infected women, women with illness episodes during pregnancy and those diagnosed with malaria in this study. Second, by the time this analysis was conducted, results for placental malaria among women from Siaya were not yet available, we could not, therefore, ascertain the positive effect of transplacental transfer in the presence of malaria infection. In addition, we could not get data for HIV antiviral therapy, adherence, or viral load among these women, although they were all under comprehensive care programme. We also used data from cohorts not necessarily designed for this study outcome. For instance, Kilifi cohort women did not have data on follow ups during pregnancy. The sample size was small for women with premature births, HIV infection, RSV infection, leading to wider confidence intervals and not so strong positive effect on predictors. However, we have provided important baseline data on the efficiency of transplacental transfer of RSV-specific antibodies in a setting where the maternal population experiences a high prevalence of malaria and HIV infections, and we have outlined some of the factors which would require mitigation or use of alternative prevention strategies during the introduction of a maternal RSV vaccine program for optimal vaccine outcome among infants.

## Conclusions

Transplacental transfer of RSV-specific antibodies among pregnant women in Kenya is efficient. Maternal characteristics differed between women from the two different geographical regions, but this did not have a significant effect on the overall transplacental transfer of RSV antibodies. Maternal immunisation in the third trimester of pregnancy as a strategy to prevent infants from severe RSV disease should be considered with other interventions which will help protect infants born prematurely<33 weeks gestation, infants with low birth weight and infants from HIV infected mothers.

## Data availability

The data is stored under restricted access and available from the authors upon request through our Data Governance Committee ( dgc@kemri-wellcome.org).

The dataset used and analysis scripts generated for this manuscript are available at Harvard Dataverse: Replication Data for: Efficiency of transplacental transfer of respiratory syncytial virus (RSV) specific antibodies among pregnant women in Kenya, https://doi.org/10.7910/DVN/XOKFFK ^24^.

This project contains the following underlying data:

- JNyiro_RSV_Antibody_Efficiency_Data_Codebook.pdf
- JNyiro_RSV_Antibody_Efficiency_Data_Readme.txt
- RSV_antibody_efficiency analysis script_04042021.do
- RSV_antibody_Efficiency_KIPMAT_MATFLU_dataset_04062021–1.tab
- RSV_antibody_Efficiency_KIPMAT_MATFLU_dataset_04062021.tab

Data are available under the terms of the Creative Commons Attribution 4.0 International license (CC-BY 4.0).

## Disclaimer

The findings and conclusions in this report are those of the author(s) and do not necessarily represent the official position of the Centers for Disease Control and Prevention. Names of specific vendors, manufacturers, or products are included for public health and informational purposes; inclusion does not imply endorsement of the vendors, manufacturers, or products by the Centers for Disease Control and Prevention or the US Department of Health and Human Services.
