# Supplementary material for: Efficiency of transplacental transfer of respiratory syncytial virus (RSV) specific antibodies among pregnant women in Kenya
Source: Wellcome Open Res. 2022 Apr 1;7:43. Originally published 2022 Feb 8. [Version 2] doi: 10.12688/wellcomeopenres.17636.2 (PMC8976186; doi:10.12688/wellcomeopenres.17636.2)
Supplement: Peer review history

# Peer review: Reviewer response for version 2

**DOI:** 10.21956/wellcomeopenres.19709.r49637

**Published:** 2022-04-01

**Authors:** Beate Kampmann

**Affiliations:** 1 The Vaccine Centre, Faculty of Infectious and Tropical Diseases, London School of Hygiene and Tropical Medicine, London, UK; 2 MRC Unit The Gambia, London School of Hygiene and Tropical Medicine, London, UK

I am satisfied with the changes made in this manuscript and it can be approved from my end.

Is the work clearly and accurately presented and does it cite the current literature?

Partly

If applicable, is the statistical analysis and its interpretation appropriate?

Partly

Are all the source data underlying the results available to ensure full reproducibility?

Yes

Is the study design appropriate and is the work technically sound?

Yes

Are the conclusions drawn adequately supported by the results?

Partly

Are sufficient details of methods and analysis provided to allow replication by others?

No

Reviewer Expertise:

Maternal immunisation, Vaccinology, Infant immunity

I confirm that I have read this submission and believe that I have an appropriate level of expertise to confirm that it is of an acceptable scientific standard.

# Peer review: Reviewer response for version 1

**DOI:** 10.21956/wellcomeopenres.19508.r48687

**Published:** 2022-03-01

**Authors:** Beate Kampmann

**Affiliations:** 1 The Vaccine Centre, Faculty of Infectious and Tropical Diseases, London School of Hygiene and Tropical Medicine, London, UK; 2 MRC Unit The Gambia, London School of Hygiene and Tropical Medicine, London, UK

The paper by Joyce Nyiro *et al. *summarises the results of a study measuring the transfer of anti-RSV antibody via the placenta in two Kenyan communities.

 The samples used stem from two unrelated cohort studies and measure neutralising RSV antibody in paired cord and maternal banked serum samples from a total of 400 mother/infant pairs, using an in-house plaque reduction neutralisation assay conducted at the KEMRI Wellcome Trust Research Programme laboratories in Kenya.

 The authors found that overall the antibody transfer was “efficient” with no significant differences between the 2 cohorts. However, HIV status and gestational age of the infant were strong predictors for reduced transplacental transfer ratios.

 The subject is important given the efforts to develop and implement an RSV vaccine targeted at pregnant women to prevent RSV infection in young infants. RSV is a known cause of significant morbidity and mortality in early life and the LEMRI team has a longstanding interest in this research area. To study transplacental antibody is therefore timely, although vaccine-induced antibody might give different results-and potentially much higher levels- than the antibody measured here which is due to natural infection.

 Conceptually, I have some questions regarding the wider interpretation of the results, as the title talks about efficiency which somewhat raises the expectation that protection from RSV disease is also addressed in this manuscript, which it is not. The results are also not interpreted in the context of potential protective efficacy or indeed should be discussed in correlation to what might be expected from the vaccine-induced antibody.

 I have made some more specific comments going through the various sections of the paper:

**Introduction:**

- The statement that neonates cannot mount protective immune responses is incorrect.
- Undernutrition is mentioned as a contributor to poor vaccine responses but not referenced-where is this evidence? Also this isn’t addressed in the manuscript.
- The rationale for using the 2 different cohorts is not provided- maybe opportunistic?
- The paper talks a lot about efficiency but a definition is not provided -more than 1.0 transfer ratio I guess is what has been defined-needs clarification.

**Methods:**

- Whilst there is more than enough description of the 2 cohorts- in fact a lot of this info is not relevant to the study aims reported here and could be moved to supplementary materials- there is a very rudimentary description of the laboratory methods.
- The sample selection is also not very clearly described as -although apparently randomly selected samples-, there is probably targeted selection for HIV+ve mothers and premature infants to address two key factors that are likely to influence the transplacental transfer ratios? Please make sure the sample selection process is very clear and state if you enriched for these subgroups as they might not have been randomly distributed across the studies.
- There is description of the RSV diagnostics which was not outlined as a research question -which might be that people wanted to ask how/if recent natural infection impacts on measurable antibody- if this was an aim it should be clearly stated. The comparison between antibody acquired from past infection and recent infection should then also be shown.
- A similar issue arises with mentioning of the infants- was an aim of the study to assess if infants who were infected with RSV had lower titres than those not? The sample size is really small to address this issue and it should be clearly stated if this was an exploratory angle or a main question.
- Both of these questions above are of interest but there is no power calculation to really address then in the current study design and it all, therefore, reads like thrown in for good measure-(or part of a doctoral thesis chapter??)

**Results:**

- The data provided are generally reassuring but their display varies between talking about PRNT titres and then suddenly switch to odds ratios- why not stick with transfer ratios which are more accessible, especially since there is next to no information on the measurements in this neutralisation assay.
- A lot of parameters are being analysed and a multi-variant analysis would be the best way to combine the read outs- HIV and preterm birth stand out-and these results align with results from other studies measuring transplacental transfer ratios for other either naturally occurring or vaccine induced transplacental antibody.
- Preterm birth and low birth weight are obviously related variables.
- Definitions for severely impaired and impaired were not introduced in the Methods section and don’t appear to be referenced- where does the definition come from and what is the clinical relevance of 0.96 versus 1.02? This needs to be discussed or at least acknowledged how we would know if/why it might make a difference- the discussion lacks depth in this area.
- Figure 2 could be better explained and the tables have far too many demographic details that do not matter- a lot of this could be moved to the supplementaries if at all of interest- the key variables that might impact on transfer are the only ones that need to be mentioned in my view- again, too much distracting detail that might come from a thesis?

The **discussion **finally touches on the differences between naturally occurring and vaccine-induced antibody but it is very short in this context- no examples of vaccine-induced antibody levels, although some of this data is publically accessible, no comparison with Palivizumab-equivalent antibody tires, no discussion about antibody not being the only correlate of protection against RSV.

 What the paper does well is to show that transplacental antibody is measurable, the transfer ratio is above 1, hence there must be active transport via nFcG receptor (not discussed!),  and that the levels can be impacted upon by HIV infection and gestational age of the infant-and this is important information given the vaccines are under advanced development, but a lot of the added detail is not positioned to address additional aspects which have however also not been clearly formulated- natural versus vaccine antibody, recent infection vs past infection in the women, any evidence of protection in the infants, and evidence/assumptions for lack of protection in infants-in context of assumed correlates of protection.

Is the work clearly and accurately presented and does it cite the current literature?

Partly

If applicable, is the statistical analysis and its interpretation appropriate?

Partly

Are all the source data underlying the results available to ensure full reproducibility?

Yes

Is the study design appropriate and is the work technically sound?

Yes

Are the conclusions drawn adequately supported by the results?

Partly

Are sufficient details of methods and analysis provided to allow replication by others?

No

Reviewer Expertise:

Maternal immunisation, Vaccinology, infant immunity

I confirm that I have read this submission and believe that I have an appropriate level of expertise to state that I do not consider it to be of an acceptable scientific standard, for reasons outlined above.

# Response

**Published:** 2022-03-25

**Authors:** Joyce Nyiro

**Affiliations:** Kemri-Wellcome Trust Research Programme, Kenya

*Thank you for the useful comments. We have addressed each of the comments below comprehensively and revised the manuscript accordingly.*

*All our responses have been provided in italic font.*

*Regarding the comment about the title of the manuscript, it is important to note that efficiency in transplacental transfer of antibodies is not the same as efficacy or effectiveness (those terms indicating protection). Our manuscript did not intend to assess antibody protection but to address the efficiency of transplacental transfer.*

**Introduction:**

 Comment:

- The statement that neonates cannot mount protective immune responses is incorrect.

*Response:*

*Thank you for picking this out. The wording has been changed to bring out the intended message. We have deleted the word “cannot” from the sentence and replaced it with the word “may not”, as a less definitive statement. However, it is well documented that very young infants may not generate a robust immune response after exposure compared to older children and adults. This has been shown to be the case with other vaccines like measles:* *https://www.ncbi.nlm.nih.gov/pmc/articles/PMC4135535/* *and https://www.frontiersin.org/articles/10.3389/fimmu.2020.595297/full.*

*In the case of RSV, infants during the neonatal period may not mount a strong immune response following infection or vaccination. It is likely the response is obscured by the presence of maternal antibodies which are decaying or might reflect an immature immune system. This phenomenon has been observed in a number of studies including a study conducted in Kilifi, Kenya by Sande et al., 2014, a study among Italian infants by Esposito et al., 2016 and in Japan in a study by Jounai et al., 2017.*

*Please see these changes in the introduction section, paragraph 2 of the revised manuscript.*

 Comment:

- Undernutrition is mentioned as a contributor to poor vaccine responses but not referenced-where is this evidence? Also this isn’t addressed in the manuscript.

*Response:*

*Thank you for the insight and we agree that this statement might not have come out clearly within the manuscript. Our mention of undernutrition in the introduction section of this manuscript was to highlight it as one of the factors which have been found to impair the transplacental transfer of antibodies. Malnutrition has been found to reduce the passive transfer of IgG antibodies from the mother to infant which reduces the efficacy of vaccines in pregnancy intended to prevent infant infection. The mechanism of malnutrition in reducing the transplacental transfer of antibodies has been related to differences in placental size, morphology, and vascular development.*

*Evidence of reduced transplacental transfer in malnourished pregnant women has been shown with* *Haemophilus influenzae type b (Hib) vaccine in Brazil* *where* *the* *vertical transmission rate was 14% lower in the malnourished pregnant group* *(Cavalcante, et al., 2008). This reference has been added in the introduction section and paragraph 4 of the manuscript revised accordingly.*

 Comment:

- The rationale for using the 2 different cohorts is not provided- maybe opportunistic?

*Response:*

*Thank you for this comment. To an extent, the selection was opportunistic as they were the only cohorts available to provide the required samples and data. In this study, we used already collected serum samples from the two existing cohorts, hence the selection of the study sites preceded the current analyses. The KIPMAT cohort was specifically set up to do this kind of analysis in relation to maternal vaccines including Group B Streptococcus (GBS) and RSV, while the Influenza surveillance cohort was set up for analysis of maternal influenza vaccine trials.*

*The choice of two cohorts instead of one cohort was purposive, in that it enabled a comparison between pregnant women from two diverse geographical regions in Kenya which fit with the main objective of the study. Additionally, one cohort was from a region known for high rates of HIV infection and the other from a region endemic for malaria which were some of the factors of interest in the transplacental transfer of antibodies. The methods section has been updated to explicitly state the rationale.*

 Comment:

- The paper talks a lot about efficiency but a definition is not provided -more than 1.0 transfer ratio I guess is what has been defined-needs clarification.

*Response:*

*We appreciate the concern raised. The definition for efficiency was provided in the statistical analysis section of this manuscript and was defined as cord to maternal RSV antibody titre ratio (CMTR) at birth. Where CMTR was one or greater than one, this was considered as normal or an efficient transfer; if CMTR was less than one, the transplacental transfer was considered as impaired. IgG antibody is actively transported across the syncytiotrophoblast cells rather than a passive diffusion process which is why we expect it to be more than 1 (Palmeira et al., 2012).*

*Since this comment has been raised multiple times, we have considered the inclusion of this definition in the introduction section of the revised manuscript for more clarity.*

**Methods:**

 Comment:

- Whilst there is more than enough description of the 2 cohorts- in fact a lot of this info is not relevant to the study aims reported here and could be moved to supplementary materials- there is a very rudimentary description of the laboratory methods.

*Response:*

*Thank you for this suggestion. We added the description of the two cohorts to provide the context of the current study and show collected metadata. We thought it was necessary to retain this information in the main manuscript following a request made by other reviewers during the process for seeking clearance of the manuscript for publication by CDC.*

*Concerning the laboratory methods, we have now provided a detailed description of the plaque reduction neutralization assay, in addition to referencing previously published procedures ( Nyiro et al., 2019). This addition has been made at the laboratory procedures section in methods of the revised manuscript in paragraphs 1 to 3.*

 Comment:

- The sample selection is also not very clearly described as -although apparently randomly selected samples-, there is probably targeted selection for HIV+ve mothers and premature infants to address two key factors that are likely to influence the transplacental transfer ratios? Please make sure the sample selection process is very clear and state if you enriched for these subgroups as they might not have been randomly distributed across the studies.

*Response:*

*Thank you for this comment and we have now clearly described the sample selection. For the Siaya cohort, we ensured adequate representation of women with HIV, malaria, known RSV infection or no recorded risk factor. In each stratum, all or a random selection of the women was conducted. This is because pregnant women with HIV, malaria and known RSV infection from the database were very few. For the Kilifi cohort, we used the random selection approach.*

*The description of the sample selection process has been updated in the Methods section of the revised manuscript.*

 Comment:

- There is description of the RSV diagnostics which was not outlined as a research question -which might be that people wanted to ask how/if recent natural infection impacts on measurable antibody- if this was an aim it should be clearly stated. The comparison between antibody acquired from past infection and recent infection should then also be shown.

*Response:*

*You are right, the question on how/if recent natural infection impacts on measurable antibodies was not part of the aim of this study. Our initial submission, before the editor’s comments, didn’t include the detailed description of the PCR methodology used in testing for RSV antigen from pregnant women who presented at the hospital for care with acute respiratory infection. However, we felt it important to include this description after a request by the journal.* *We have therefore retained this description in the main manuscript because removing it might conflict with the journal requirements.*

 Comment:

- A similar issue arises with mentioning of the infants- was an aim of the study to assess if infants who were infected with RSV had lower titres than those not? The sample size is really small to address this issue and it should be clearly stated if this was an exploratory angle or a main question.

*Response:*

*Thank you for this comment. This section has now been deleted from the manuscript due to a similar comment by the first reviewer. Again, this was not part of the objectives of this study but an exploratory analysis.*

**

 Comment: *​​​​​​​*

- Both of these questions above are of interest but there is no power calculation to really address then in the current study design and it all, therefore, reads like thrown in for good measure-(or part of a doctoral thesis chapter??)

*Response:*

*Thank you for this comment. We agree we didn’t include power calculation in this manuscript because we didn’t find its utility. However, we had conducted power calculation in the design of the study to assess the degree of reduction of transplacental transfer by any of the covariates. The power calculation applied reduced transplacental transfer by* *malaria and hypergammaglobulinaemia for RSV specific antibodies of 58% and 90% respectively which was observed in the Gambia (Okoko et al., 2001).* *We had estimated a sample of 160 pregnant women with past RSV infection in our study to* *have 82.8% power to* *detect a 12.5% reduction in transplacental transfer of RSV specific antibodies by any of the covariates/factors such as* *anaemia, malaria, HIV status, maternal age and parity. However, due to the small sample size for women with the different characteristics of HIV infection, anaemia, and other illnesses, the study only focused on the sample size estimation for the primary objective which was to determine the cord to maternal titre ratio as described in this manuscript, and using this data explored the effect of covariates. The lack of adequate sample size for each group was addressed in the limitation section of this manuscript.*

**Results:**

 Comment: **​​​​​​​**

 The data provided are generally reassuring but their display varies between talking about PRNT titres and then suddenly switch to odds ratios- why not stick with transfer ratios which are more accessible, especially since there is next to no information on the measurements in this neutralisation assay.

*Response:*

*Thank you for this comment. To clarify further, the results in this manuscript also present the distribution of PRNT titres from maternal and cord blood samples (Figure 1). These PRNT titres have been used to calculate the cord to maternal RSV antibody titre ratios (CMTR) which were used to define the efficiency of the RSV antibody transplacental transfer. The transplacental transfer efficiency was then used as a binary outcome (normal vs impaired transfer) in a multivariable logistic regression model to determine the effect of covariates.*

*These details have been described in the methods, statistical analysis section and also shown in the results section of this manuscript.*

 Comment:

- A lot of parameters are being analysed and a multi-variant analysis would be the best way to combine the read outs- HIV and preterm birth stand out-and these results align with results from other studies measuring transplacental transfer ratios for other either naturally occurring or vaccine induced transplacental antibody.

*Response:*

*Thank you and we agree with this comment. This is what we did in our analysis. These details are shown in the statistical analysis section and the data analysis script provided with the replication dataset of this manuscript.*

 Comment:

- Preterm birth and low birth weight are obviously related variables.

*Response:*

*We agree with this statement. However, whereas preterm infants born before 37 weeks of gestation normally have low birth weight, an infant can also have low birth weight even if they are born at full term (>37weeks).*

**

 Comment:​​​​​​​ *​​​​​​​*

- Definitions for severely impaired and impaired were not introduced in the Methods section and don’t appear to be referenced- where does the definition come from and what is the clinical relevance of 0.96 versus 1.02? This needs to be discussed or at least acknowledged how we would know if/why it might make a difference- the discussion lacks depth in this area.

*Response:*

*Thank you for this comment. All definitions of transplacental efficiency were provided in the statistical analysis (methods) section of this manuscript. The efficient transplacental transfer was defined as CMTR>1 and an impaired transplacental transfer as CMTR<1. A CMTR of 0.96 is less than one meaning transplacental transfer is impaired while a CMTR of 1.02 is greater than one meaning transplacental transfer is normal/ efficient. This definition and analysis have already been used by the following studies: Australia (Nusrat, H. et al,. 2022), in Papua New Guinea (Atwell, JE. et al., 2016) and in Nepal (Chu, H. et al., 2017)*

 Comment:​​​​​​​

- Figure 2 could be better explained and the tables have far too many demographic details that do not matter- a lot of this could be moved to the supplementaries if at all of interest- the key variables that might impact on transfer are the only ones that need to be mentioned in my view- again, too much distracting detail that might come from a thesis?

*Response:*

*This has been well noted and Figure 2 has been removed from the main manuscript and is now available together with the replication dataset of this manuscript as Supplementary File 2. The tables have been revised as suggested and Table 2 has also been moved to the replication dataset as Supplementary File 1.*

 Comment:

 The **discussion **finally touches on the differences between naturally occurring and vaccine-induced antibody but it is very short in this context- no examples of vaccine-induced antibody levels, although some of this data is publically accessible, no comparison with Palivizumab-equivalent antibody tires, no discussion about antibody not being the only correlate of protection against RSV.

 What the paper does well is to show that transplacental antibody is measurable, the transfer ratio is above 1, hence there must be active transport via nFcG receptor (not discussed!),  and that the levels can be impacted upon by HIV infection and gestational age of the infant-and this is important information given the vaccines are under advanced development, but a lot of the added detail is not positioned to address additional aspects which have however also not been clearly formulated- natural versus vaccine antibody, recent infection vs past infection in the women, any evidence of protection in the infants, and evidence/assumptions for lack of protection in infants-in context of assumed correlates of protection.

*Response:*

*Thank you for the useful comments and input into this manuscript*. *We have considered your concerns and have done the following changes in the manuscript based on your suggestions and where we thought the data available was not sufficient to address some of those aspects.*

 1. *The aspect of natural versus vaccine antibody,*

*We agree this would have been worth discussing in this manuscript since the nature and quality of natural antibody is different from that of vaccine antibody. However, this was not part of the study objectives and was not supported by the available data. Our manuscript only focused on the transplacental transfer of IgG antibodies and the factors likely to impact this transfer. The section in the discussion which had highlighted this aspect has been deleted.*

*2. Recent infection versus past infection*

*We acknowledge that this aspect didn’t come out very clearly in the earlier version of the manuscript. This was not part of the study objectives. However, methodologies for testing of recent infections using PCR were only provided in the manuscript to describe the procedure used to identify women with RSV related acute respiratory infection who presented for care at the hospital. In this study, we did not measure antibodies due to recent infection and that’s why it is not discussed in this manuscript. Since inclusion of the PCR laboratory procedure was a request by the journal editor, we have retained this section in the main manuscript.*

*3. Evidence of protection in infants* *and evidence/assumptions for lack of protection in infants*

*We agree we didn’t explicitly explain this aspect in the manuscript. This is because, it was not an objective of the study, and the data could not support this analysis. Paragraph 3 in the discussion which had mentioned this aspect has been deleted from this manuscript.*

*Since these aspects are of high interest, we would like to mention that, there are other studies conducted in Kilifi, Kenya which previously sought to address some of these questions. These include Sande et al., 2014 (Recent infection versus past infection) and Nyiro et al., 2016 (evidence of protection by maternal antibody from natural RSV infection).*​​

# Peer review: Reviewer response for version 1

**DOI:** 10.21956/wellcomeopenres.19508.r48576

**Published:** 2022-02-15

**Authors:** Kondwani Charles Jambo

**Affiliations:** 1 Malawi-Liverpool-Wellcome Trust Clinical Research Programme, University of Malawi College of Medicine, Blantyre, Malawi

The authors conduct a serological cohort study in Kenya where they study the transplacental transfer of RSV-specific antibodies. They report the efficient transplacental transfer of RSV-specific antibodies among the cohort. They observe that prematurity (<33 weeks gestation), HIV infection, and low birth weight as key factors associated with reduced efficiency of transplacental transfer. They conclude that there is an efficient transplacental transfer of RSV-specific antibodies among pregnant women in Kenya and recommend the integration of other preventive interventions with maternal RSV vaccination targeting infants born premature, low birth weight, and HIV-infected mothers.

 This is a timely study in the field as there is a lot of traction on maternal RSV vaccination. The study identifies key groups that may not maximally benefit from maternal RSV vaccination due to inefficient transplacental transfer of RSV-specific antibodies from mother to child. It has important implications on future RSV vaccination policy as it suggests that extra interventions may be needed for children born to HIV-infected mothers, low birth weight babies, and infants born premature.

 Overall, this study is well-conducted with most of the conclusions supported by the presented data. However, I recommend that the authors remove paragraph 3 from the discussion section as the concluding statement is not supported by their data.

Is the work clearly and accurately presented and does it cite the current literature?

Yes

If applicable, is the statistical analysis and its interpretation appropriate?

Yes

Are all the source data underlying the results available to ensure full reproducibility?

Yes

Is the study design appropriate and is the work technically sound?

Yes

Are the conclusions drawn adequately supported by the results?

Yes

Are sufficient details of methods and analysis provided to allow replication by others?

Yes

Reviewer Expertise:

Immunology

I confirm that I have read this submission and believe that I have an appropriate level of expertise to confirm that it is of an acceptable scientific standard.

# Response

**Published:** 2022-03-24

**Authors:** Joyce Nyiro

**Affiliations:** Kemri-Wellcome Trust Research Programme, Kenya

*Thank you for your comments and positive feedback on our manuscript. The referred paragraph 3 in the manuscript’s discussion section has now been deleted.*
